# The ERK Signaling Cascade Inhibits Gonadotropin-Stimulated Steroidogenesis

**DOI:** 10.26502/jbb.2642-91280066

**Published:** 2023-01-13

**Authors:** Rony Seger, Tamar Hanoch, Revital Rosenberg, Ada Dantes, Wolfgang E. Merz, Jerome F. Strauss, Abraham Amsterdam

**Affiliations:** 1 Department of Immunology and Regenerative Biology, The Weizmann Institute of Science, Rehovot 7160001, Israel; 2 Department of Molecular Cell Biology, The Weizmann Institute of Science, Rehovot 7160001, Israel; 3 Biochemie-Zentrum Heidelberg, University of Heidelberg, Im Neuenheimer Feld 328, 69120 Heidelberg, Germany; 4 Center for research on Reproduction and Women’s Health, University of Pennsylvania, Philadelphia, USA

**Keywords:** LH, FSH, PKA, ERK, MEK, StAR

## Abstract

The response of granulosa cells to Luteinizing Hormone (LH) and Follicle- Stimulating Hormone (FSH) is mediated mainly by cAMP/protein kinase A (PKA) signaling. Notably, the activity of the extracellular signal-regulated kinase (ERK) signaling cascade is elevated in response to these stimuli as well. We studied the involvement of the ERK cascade in LH- and FSH-induced steroidogenesis in two granulosa-derived cell lines, rLHR-4 and rFSHR-17, respectively. We found that stimulation of these cells with the appropriate gonadotropin induced ERK activation as well as progesterone production downstream of PKA. Inhibition of ERK activity enhanced gonadotropin-stimulated progesterone production, which was correlated with increased expression of the Steroidogenic Acute Regulatory Protein (StAR), a key regulator of progesterone synthesis. Therefore, it is likely that gonadotropin-stimulated progesterone formation is regulated by a pathway that includes PKA and StAR, and this process is down-regulated by ERK, due to attenuation of StAR expression. Our results suggest that activation of PKA signaling by gonadotropins not only induces steroidogenesis but also activates down-regulation machinery involving the ERK cascade. The activation of ERK by gonadotropins as well as by other agents may be a key mechanism for the modulation of gonadotropin-induced steroidogenesis.

## Introduction

Gonadotropic hormones, Follicle Stimulating Hormone (FSH) and Luteinizing Hormone (LH), which are released from the pituitary, play a crucial role in controlling reproductive functions in males and females. The pleotropic effects of gonadotropins are manifested in various cells of the reproductive system including LH and FSH in ovarian granulosa cells, LH in theca interna cells, FSH in testicular Sertoli cells, and LH in Leydig cells [1,2,3]. One of the main effects of both LH and FSH on the ovary is stimulation of the production of estradiol and progesterone, which play important roles in ovarian function and control of the reproductive cycle (reviewed in [4]). The mechanisms involved in the regulation of progesterone production by ovarian granulosa cells have been characterized in detail. Gonadotropins exert their stimulatory activity via interaction with specific seven-transmembrane receptors, being the LH-receptor and FSH-receptor. Upon binding of the gonadotropins, both receptors stimulate the Gs protein, which activates the membrane-associated adenylyl cyclase, causing an elevation of intracellular cAMP [5]. This cyclic nucleotide serves as a second messenger for the upregulation of the steroidogenic acute regulatory protein (StAR) and the cytochrome P450 (P450scc) enzyme system (reviewed in [6,7]. Activation of alternative signaling pathways by the gonadotropin receptors was described as well, including calcium ion mobilization, activation of the phosphoinositol pathway, and stimulation of chloride ion influx (reviewed in [8]). However, these gonadotropin-induced signaling processes were not previously implicated in the modulation of steroidogenesis [5]. Another process that plays an important role in inhibiting gonadotropin-induced steroidogenesis is the desensitization of the gonadotropin receptor [2]. G-protein coupled receptor kinase phosphorylation of the gonadotropin receptors, the adaptor protein β-arrestin and massive internalization of the receptors are thought to play a role in the downregulation of gonadotropin signaling. However, since desensitization precedes the internalization of the gonadotropin receptors [9], additional mechanisms are likely to participate in the rapid attenuation of gonadotropin signals downstream of the receptors.

The Extracellular Signal-Regulated Kinases (ERK) include three kinases (p42ERK2, p44ERK1, p42ERK1b/c) that belong to the family of the signaling Mitogen-Activated Protein Kinases (MAPKs). Upon extracellular stimulation, the ERKs are activated by a network of interacting proteins, which funnel the signals into a multi-tier kinase cascade (reviewed in [10,11]). The activated ERK in turn regulate additional signaling kinases (e.g., RSK) or can by themselves phosphorylate and activate target regulatory proteins (e.g., Elk1), which govern various cellular processes. Although the ERK were first implicated in the regulation of proliferation and differentiation, it is presently known that these kinases participate also in the control of cellular morphology, learning and memory in the CNS, apoptosis and carcinogenesis [10]. It has previously been shown that ovarian granulosa cell ERK is activated (2-5-fold) in response to LH and FSH [12, 13]. These effects were mimicked by elevation of intracellular cAMP, and the FSH effect was inhibited by inhibitors of PKA, indicating that ERK transduces signals downstream of PKA in gonadotropin-induced granulosa cells. In the present work, we show that gonadotropins induce ERK activation and progesterone production via cAMP in immortalized granulosa cell lines. These cell lines are homogeneous populations, unlike follicular granulosa cells which represent a heterogeneous population with respect to LH receptor content and the degree of maturation [14]. Interestingly, inhibition of ERK activation causes an elevation in gonadotropin-cAMP-induced progesterone production, while activation of ERK inhibits this process. Moreover, the addition of a MEK inhibitor that diminishes ERK activity elevated the intracellular content of StAR, which operates downstream of cAMP, suggesting that the inhibitory effect of the ERK on steroidogenesis may be mediated by the reduction in the expression of StAR. Therefore, it is likely that gonadotropin-induced progesterone formation is regulated by PKA, which induces not only the expression of StAR, but also a counteracting down-regulating mechanism. These two mechanisms are simultaneously brought into play by the activation of ERK, which reduces StAR expression.

## 2. Material and Methods

### 2.1. Stimulants, inhibitors antibodies and other reagents

Human FSH (hFSH) Human LH (hLH) and Human Chorionic Gonadotropin (hCG) were kindly provided by the NIH and Dr. Parlow. Deglycosilated hCG was enzymatically prepared as previously described [15]. Mouse monoclonal anti-diphospho ERK (anti-active ERK/MAPK) antibodies (DP-ERK Ab), and anti-general ERK antibody were obtained from Sigma, Israel (Rehovot, Israel). Anti-C-terminal ERK1 antibody (C16) was purchased from Santa Cruz. Polyclonal antibodies to human StAR were raised in rabbit [16]. Alkaline phosphatase, horseradish peroxidase and flourescein conjugated secondary antibodies were purchased from Jackson ImmunoResearch laboratories Inc. (West Grove, Pennsylvania). PD98059 and U0126 were purchased from Calbiochem (San Diego). H89, Forskolin, 8-Br-cAMP were obtained from Sigma (St. Louis).

### 2.2. Cell lines

rLHR-4 cell line was established by cotransfection of rat preovulatory granulosa with mutated p53 (Val135-p53) Ha-ras genes and plasmid expressing the rat LH/CG-receptor [17]. The rFSHR-17 cell line was established by immortalization of preovulatory rat granulosa cells via cotransfection of primary cells with SV40 DNA and an HA-ras gene. Cells were transfected with plasmid expressing the rat FSH receptor [18]. The cells were maintained in F12/DMEM medium (1:1) containing 5% fetal calf serum.

### 2.3. Stimulation and harvesting of cells

Subconfluent cultures were serum-starved for 16 h, and subsequently incubated for selected time intervals with the indicated agents in the presence or absence of various inhibitors. Following stimulation, cells were washed twice with ice-cold phosphate buffered saline, once with Buffer A (50 mM -glycerophosphate, pH 7.3, 1.5 mM EGTA, 1 mM EDTA, 1 mM DTT, and 0.1 mM sodium vanadate, [19]), and were subsequently harvested in ice-cold Buffer A + proteinase inhibitors [19]. Cell lysates were centrifuged at (20,000xg, 20 min). The supernatant was assayed for protein content and subjected to a Western blot analysis or to immunoprecipitation as below. For the detection of StAR protein, cells were lysed in RIPA Buffer [19] and subjected to Western blot analysis.

### 2.4. Transfection of PKI and ERK plasmids into cells

The rLHR-4 and rFSHR-17 cells were grown in Dulbecco’s modified Eagle’s medium (DMEM) supplemented with 10% fetal calf serum (FCS) up to 70% confluency. The plasmids used were RSV-PKI and RSV-PKI mutant, [20] (a generous gift from Dr. R.A.Maurer, Oregon Health Sciences University, Portland), and pGFP-ERK2 [21]. The plasmids were introduced into the two cell types using Lipofectamine (Gibco-BRL) according to the manufacturer instruction. About 15-20% transfection was observed in the two cell lines using a Zeiss florescent microscope. After transfection, the rLHR-4 cells were grown in DMEM+10% FCS for 6 h and then starved in DMEM+0.1% fetal calf serum for additional 14 h. The rFSHR- 17 cells were grown in DMEM+10% FCS for 20 h. The transfected cells were then stimulated and harvested as above.

### 2.5. Western Blot Analysis

Cell supernatants, which contained cytosolic proteins were collected, and aliquots from each sample (30 μg) were separated on 10% SDS-PAGE followed by Western blotting with the appropriate antibodies. Alternatively, immunoprecipitated proteins were boiled in sample buffer and subjected to SDS-PAGE and Western blotting. The blots were developed with alkaline phosphatase or horseradish peroxidase-conjugated anti- mouse or rabbit antibodies.

### 2.6. Determination of ERK activity by phosphorylation

Cell supernatants (200 μg proteins) were subjected to immunoprecipitation with monoclonal anti-ERK C-terminal antibodies (C16, Santa Cruz) as described above. During the final step of immunoprecipitation, pellets were washed with buffer A, resuspended in 15 μl of buffer A, and incubated (20 min, 30°C) with 5 μl of 2 mg/ml myelin basic protein (MBP) and 10 μl of 3x reaction mix (30 mM MgCl2, 4.5 mM DTT, 75 mM β-glycerophosphate, pH 7.3, 0.15 mM Na3VO4, 3.75 mM EGTA, 30 μM calmidazolium, 2.5 mg/ml bovine serum albumin and 100 μM [γ^32^P]-ATP (2 cpm/fmol)). The phosphorylation reactions were terminated by addition of sample buffer and boiling (5 min) and the samples were analyzed by SDS-PAGE and autoradiography as previously described [19].

### 2.7. Progesterone assay

Progesterone secreted into the culture medium was assayed by radioimmunoassay as previously described [22].

### 2.8. Localization of StAR protein by immunofluorescence

Cells were cultured on 24x24 mm cover glasses placed in 35 mm plastic tissue culture dishes. Cells were fixed with 3% paraformaldehyde subsequent to 24 h incubation at 37°C with the appropriate stimulants and visualized in a Zeiss florescent microscope following incubation with 1:200 dilution of antiserum to human StAR and goat anti-rabbit antibodies conjugated to floresceine. For negative controls, cells were incubated normal rabbit serum followed by the second antibodies.

## 3. Results

Stimulation of granulosa cells with the gonadotropins LH or FSH induces several cellular processes, including de-novo synthesis of steroid hormones. In order to study the signaling pathways that couples gonadotropin receptors to the regulation of progesterone production, we used two distinct granulosa cell lines expressing either LH/CG or FSH receptors: rLHR-4 and rFSHR-17. Addition of the appropriate gonadotropins to these cells has previously been shown to stimulated cAMP production, activation of PKA and induction of steroidogenesis [18] and data not shown). Since the ERK cascade was implicated in the signaling of G protein-coupled receptors [23], we first examined whether the ERK cascade is also activated in the rLHR-4 and rFSHR-17 cell lines.

### 3.1. Activation of ERK by hCG, Deglycosylated hCG (dghCG) and cAMP in rLHR-4 cells.

Serum-starved rLHR-4 cells were stimulated with hCG, which signals via the LH receptor [2], and phosphorylation of the activation TEY motif of ERK was then assessed using a Western blot analysis with DP-ERK Ab [24]. Considerable staining of three bands at 42, 44 and 46 kDa (ERK2, ERK1 and ERK1b respectively [19]) was detected in the resting, non-stimulated, cells. The intensity of staining of ERK2 and ERK1 was enhanced (~5-fold) 5-20 min after the addition of hCG, and remained high (~3-fold) up to 60 min after stimulation. The appearance of p46 ERK1b is of particular interest because although ERK1b has been reported to exist in rat [25], its abundance and relative activity as compared to that of ERK1 and ERK2 are usually small. Interestingly, the basal activity of ERK1b in rLHR-4 cells was as high as that of ERK1, only modestly increased 5-20 min after stimulation (~2-fold), and declined to basal level 40 min later. The kinetics, which are different from that of ERK1 and ERK2, suggests a differential mode of ERK1b-regulation as recently demonstrated in EJ cells [19]. We next examined LH, which like hCG, specifically acts via the LH/CG-receptors.

**Figure 1:**
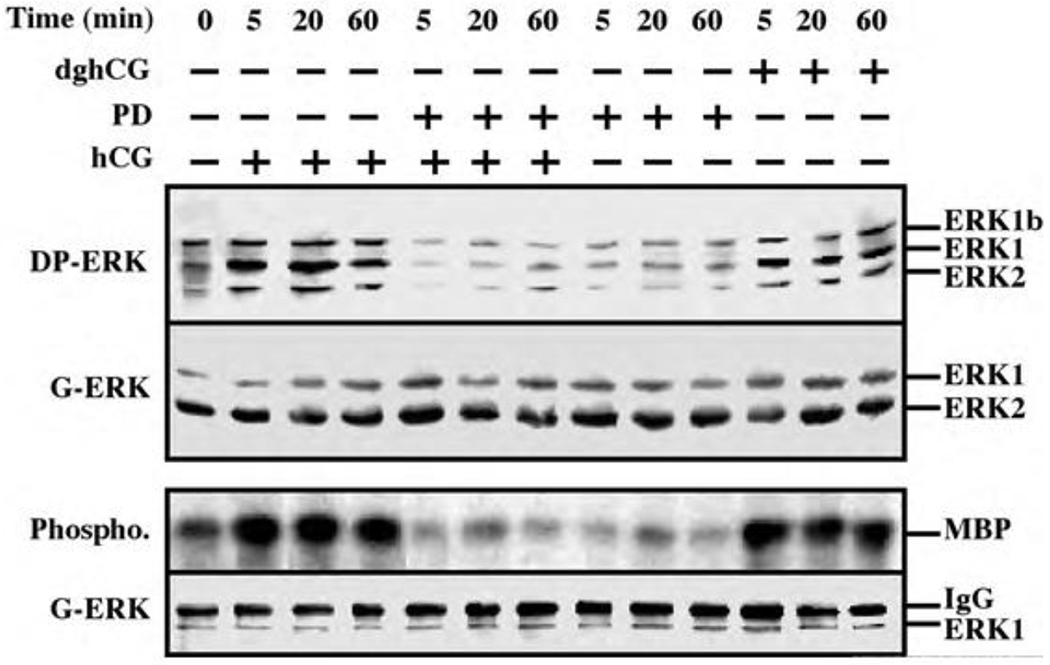
Activation of ERK/MAPK by hCG and dghCG in rLHR-4 cells. rLHR-4 cells were serum starved for 16 h, and then stimulated with hCG (3 iu/ml) with or without PD98059 (PD, 15 min pre stimulation, 25 μM), with PD98059 (25 μM) alone or with dghCG (3 iu/ml) for the indicated times. Cytosolic extracts (50 μg) were subjected to immunoblotting with DP-ERK (upper panel) or with anti-general ERK antibody (G-ERK, second panel). Alternatively, the cytosolic extracts were subjected to immunoprecipitation with anti-C terminal ERK1 antibody (C16) followed by *in vitro* phosphorylation of MBP as described under Material and Methods (3^rd^ panel, Phospho.). The amount of immunoprecipitated ERK for the phosphorylation reaction was determined by Western blotting with the anti-general ERK antibody (bottom panel). The position of ERK2, ERK1 and ERK1b, MBP and IgG is indicated. Each of these experiments was reproduced at least three times.

The effect of LH on ERK activity was essentially the same as that of hCG under all conditions examined (data not shown). Deglycosylated hCG (dghCG), which has previously been reported to maintain the same affinity for binding to the LH-receptor as the intact hormone but retains only a residual activity for stimulation of steroidogenesis [26], also caused activation of ERK. However, this activation was significantly lower than that achieved by the intact hormone (2.5-fold activation 20 min after dghCG treatment as compared to 4.5-fold 20 min after hCG treatment (Figure 1)). Since LH and hCG have previously shown to transmit their signal via Gs and cAMP [26], we examined the role of cAMP-elevating agents on the ERK activity. Indeed, both 8-Br-cAMP, and forskolin, which activates adenylyl cyclase, significantly activated ERK phosphorylation in the rLHR-4 cells (data not shown), indicating that the hCG-induced ERK activation may be dependent on elevated cAMP. Besides ERK phosphorylation of the TEY motif, which mainly reflects MEK activity, we also measured the activity of ERK itself. This was performed by immunoprecipitation with anti-C-terminal ERK1 antibody followed by phosphorylation of the general substrate - myelin basic protein (MBP [19]). As expected, this method revealed that the activity of ERK correlated well with the regulatory phosphorylation of ERK (Figure 1, bottom two panels), verifying that both hCG and dghCG cause a 4-5-fold activation of ERK1 activity in rLHR-4 cells. Addition of the MEK inhibitor, PD98059, reduced both hCG-stimulated and non- stimulated activity of ERK to below basal levels, and a similar reduction was observed for dghCG-, forskolin- and 8-Br-cAMP- stimulated activity of ERK (Figure 1 and data not shown). None of the treatments caused any significant change in the total amount of the ERKs as judged by staining with an anti-general ERK antibody (7884) which recognizes both ERK1 and ERK2 much better than ERK1b (Figure 1; G-ERK).

### 3.2. Activation of ERK by FSH and cAMP in rFSHR-17 cells

**Figure 2:**
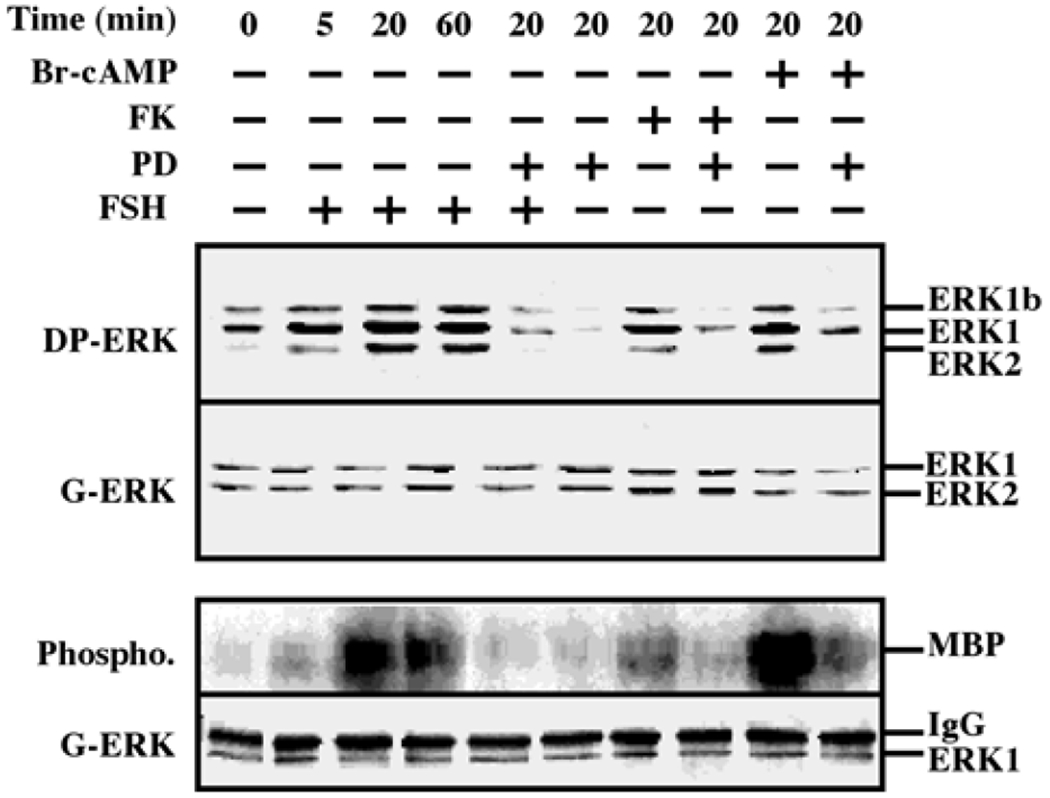
Activation of ERK/MAPK by FSH/cAMP in rFSHR-17 cells. RFSHR-17 cells were serum-starved for 16 h and then stimulated with FSH (3 iu/ml) with forskolin (FK, 50 μM), with 8-Br-cAMP (Br-cAMP, 50 μM) with or without PD98059 (PD, 15 min pre-stimulation, 25 μM), or with PD98059 (25 μM) alone for the indicated times. Cytosolic extracts (50 μg) were subjected to immunoblotting with DP-ERK (upper panel) or with anti-general ERK antibody (G-ERK, second panel). Alternatively, the cytosolic extracts were subjected to immunoprecipitation with anti-C terminal ERK1 antibody (C16) followed by *in-vitro* phosphorylation of MBP as described under Material and methods (phospho, 3^rd^ panel). The amount of immunoprecipitated ERK for the phosphorylation reaction was determined by Western blot with the anti-general ERK antibody (bottom panel). The position of ERK2, ERK1 and ERK1b, MBP and IgG is indicated. Each of these experiments was reproduced at least three times.

We tested the ability of FSH to stimulate ERK activity in the rat granulosa-derived cell line, rFSHR-17. Similarly to the rLHR-4 line, there was considerable staining of all three ERK isoforms, ERK2, ERK1 and ERK1b, in Western blots of extracts of serum-starved cells. This staining was enhanced by the addition of FSH to the cells, in kinetics that were slightly slower than the kinetics of hCG stimulation in rLHR-4 cells (Figure 2 upper lanes). The staining of the three ERK isoforms was enhanced 5 min after FSH stimulation, peaked (5-fold above basal level) at 20 min. after stimulation and slightly decreased at 60 min. Also in these cells, the cAMP stimulating agents, forskolin and 8-Br-cAMP, enhanced the phosphorylation of the three ERK isoforms (3- and 5-fold above basal level, respectively). None of the treatments caused any change in the amount of the ERK isoforms as judged by the staining with a general anti-general ERK antibody, confirming that as for the hCG experiment, the changes detected by the DP-ERK Ab are indeed due to changes in ERK phosphorylation and not due to induction of ERK expression. In addition, we examined ERK activity by immunoprecipitation and phosphorylation of MBP. We found (Figure 2, bottom) that not only ERK phosphorylation but also ERK activity was stimulated by FSH, forskolin and 8-Br-cAMP (and reduced by PD98059), confirming that both LH and FSH receptors can transmit signals to the ERK pathway in the examined cells.

### 3.3. PD98059 stimulates FSH and hCG-induced steroidogenesis

**Figure 3:**
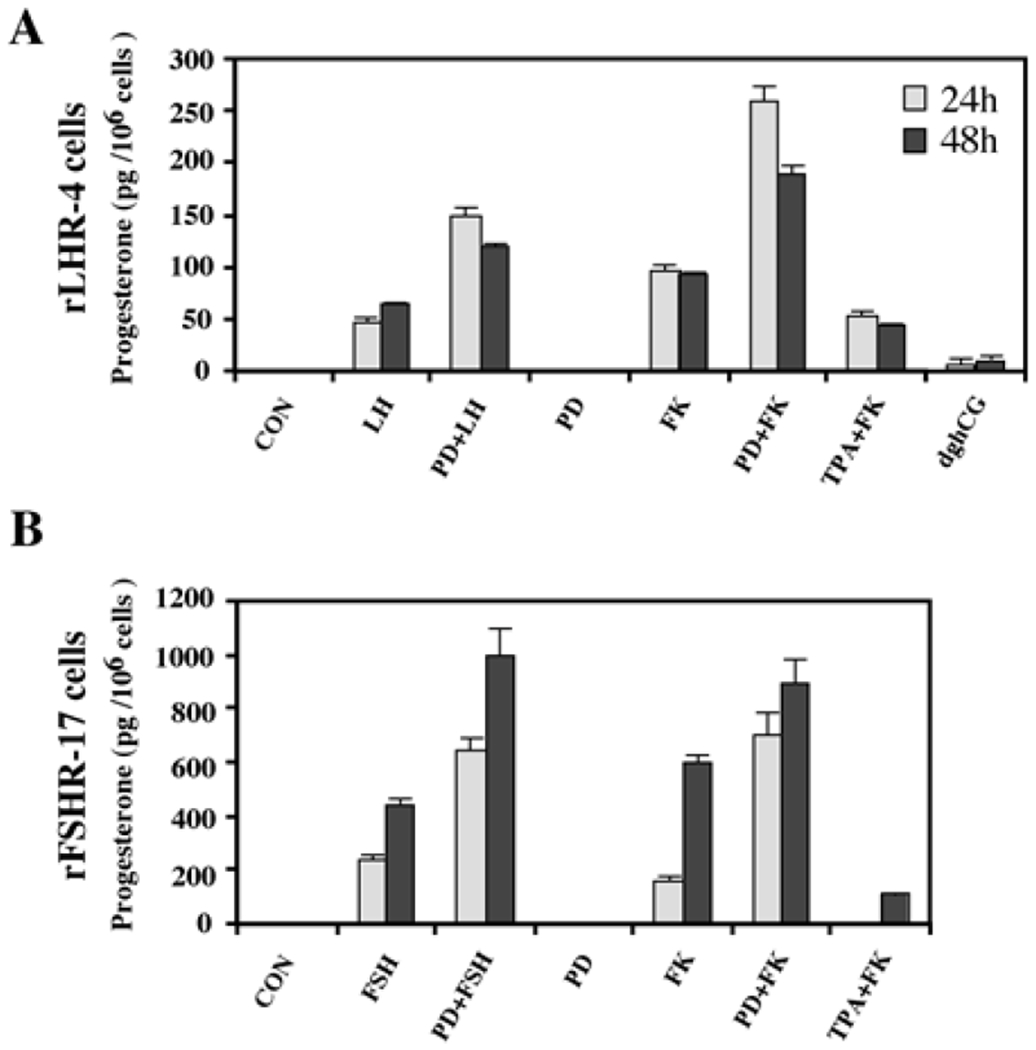
Enhancement of progesterone production by MEK inhibitor, gonadotropins and cAMP-stimulated rLHR-4 and rFSHR-17 cells. Subconfluent cultures were treated with PD98059 alone (PD, 25 μM), hCG (3 iu/ml), hFSH (3 iu/ml) dghCG (3 iu/ml), forskolin (FK, 50 μM) TPA (100 nM) or the same reagent with PD98059 as indicated for 24 h or 48 h, after which progesterone production was determined as described in Materials and Methods. Data are means of triplicate +/-standard error. These experiments were repeated four times.

One of the important cellular processes that are stimulated by gonadotropins in granulosa cells is steroidogenesis [27]. Indeed, a significant increase in progesterone production was observed 24 and 48 h after LH stimulation of rLHR-4 cell line (Figure 3A). hCG had a similar effect to that of LH (data not shown), while dghCG had a very small effect, and forskolin caused a two-fold greater induction of progesterone production than LH. In order to examine whether the activated MAPK cascade is also involved in the induction of progesterone production, we incubated the rLHR-4 cells with the MEK inhibitor, PD98059. This inhibitor had no effect by itself on progesterone production by rLHR-4 cells. However, when the cells were incubated with PD98059 for 15 min prior to LH induction there was a 3-fold increase in LH-induced progesterone production (Figure 3), under conditions where ERK activity was completely abolished (Figure 1). A Similar stimulatory effect on progesterone production was observed when the MEK inhibitor was added prior to stimulation of the cells with forskolin (Figure 3), hCG, and 8-Br-cAMP (data not shown). Similar to the rLHR-4 cells, MEK inhibitor dramatically increased steroidogenesis in rFSHR-17 cells. Thus, in these cells FSH and forskolin caused a significant elevation of progesterone production after 24 and 48 h, which was dramatically amplified by the addition of PD98059. In contrast to the induction by the MEK inhibitor, TPA, which is a known activator of the ERK cascade [11] had a negative effect on the forskolin-induced production of progesterone in both cell lines after 24 and 48 h. Taken together, these results suggest that the ERK signaling cascade suppress gonadotropin-stimulated progesterone production.

### 3.4. MEK inhibitors stimulate expression of StAR

**Figure 4:**
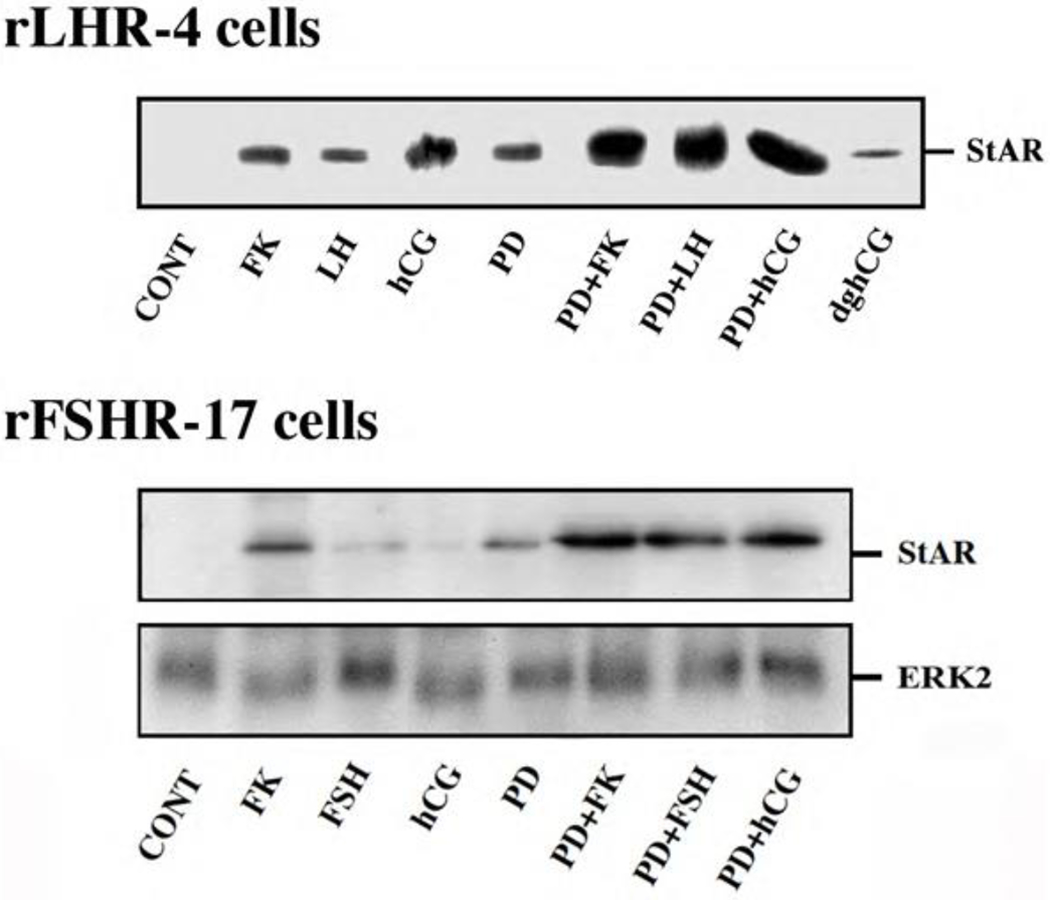
Expression of StAR in rLHR-4 and rFSHR-17 cells. Subconfluent cultures were stimulated with forskolin (FK, 50 μM), PD98059 (PD, 25 μM), hCG (3 iu/ml) hFSH (3 iu/ml), dghCG (3 iu/ml), hLH (3 iu/ml) or combination of them as indicated for 24 h. Then, the cells were extracted as described under Materials and Methods and the extracts were subjected to SDS-PAGE and Western blotting using anti-StAR antibody. The arrow indicates mature StAR protein at 34 kDa. These experiments were repeated three times

Steroidogenic acute regulatory (StAR) protein plays a crucial role in the regulation of cholesterol transport from the outer to the inner mitochondrial membrane, where cytochrome P450scc participates as a rate-limiting enzyme in steroidogenesis, conversion cholesterol into pregnenolone [28]. The induction of StAR and its downstream effects are likely to be cAMP-dependent processes as reported for gonadotropin induced steroidogenesis in the gonads and ACTH-stimulated steroidogenesis in the fasciculata cells of the adrenal [28]. Moreover, since StAR protein is known to have a short functional half-life [29], we studied whether downregulation of StAR may explain the effect of the ERK cascade on progesterone production. Thus, rLHR-4 cells were treated with the various agents and examined for the expression of StAR 24 h after stimulation. As expected, LH, hCG, forskolin and to a considerably lesser extent dghCG induced the expression of StAR under the conditions examined (Figure 4). PD98059 alone caused an induction of StAR by itself, but when the cells where pre-incubated with this MEK inhibitor prior to the addition of forskolin, LH and hCG there was a synergistic elevation in the production of StAR. Similar results were obtained also in the rFSHR-17 cells where PD98059 dramatically increased the forskolin-and FSH- induced expression of StAR. Thus, the ERK cascade may negatively regulate steroidogenesis, and this can be explained by the attenuation of StAR expression, which may be the regulatory component that integrates the signals from both the cAMP and the ERK pathway to regulate the rate of steroidogenesis.

**Figure 5:**
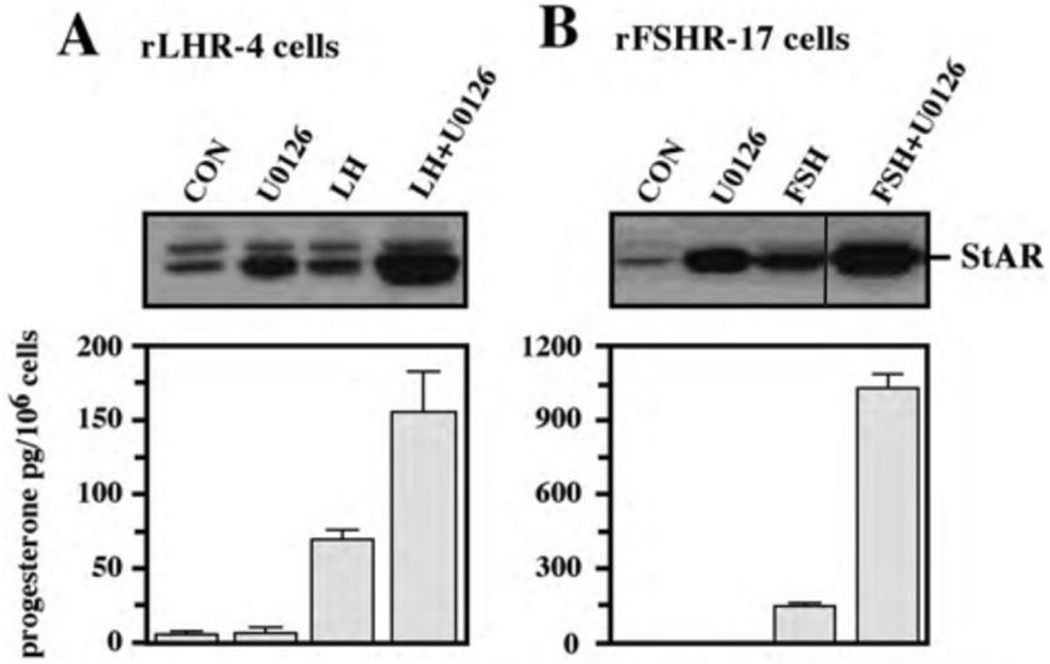
Effect of U0126 on StAR expression and progesterone production. Subconfluent cultures of either rLHR-4 (A) or rFSHR-17 (B) cells were stimulated for 24 h with LH (3 iu/ml, A) hFSH (3 iu/ml, B), U0126 (10 μM) or combination of the gonadotropins with U0126 in the same concentrations. Expression of StAR (upper panel) and progesterone production (lower panel) were detected as described above.

To further verify the results obtained with PD98059 we used an additional specific MEK inhibitor, the U0126 [30]. As observed with the PD98059, addition of this inhibitor to both rLHR-4 and rFSHR-17 cells caused an elevation in the amount of 30 kDa mature StAR [31] within 24 h (Figure 5). Addition of the gonadotropins alone also elevated this expression, but when the inhibitor was added together with the appropriate gonadotropins, the expression of StAR was significantly higher and reached up to 10-fold above basal expression levels. This was significantly higher compared to the amounts expected from the expression induced by U0126 and gonadotropin alone. Interestingly, in some of the experiments, a 37 kDa pre-StAR [31] was detected by the anti-StAR antibody (Figure 5A). This cytosolic protein is known to be maintained in a low steady state level because it rapidly matures into the 30 kDa form of StAR in the mitochondria [31]. Unlike the 30 kDa StAR, the relative low amount of this 37 kDa protein did not change upon LH, FSH or MEK inhibitors (Figure 5). We then studied the effect of U0126 on steroidogenesis in the rLHR-4 and the rFSHR-17 cells. Similar to the results of PD98059, U0126 did not induce steroidogenesis by itself but synergised with the gonadotropins to produce high amounts of progesterone (Figure 5).

Taken together, our results indicate that MEK inhibitors dramatically increase gonadotropin-induced StAR expression and steroidogenesis. However, the MEK inhibitors themselves induced clear elevation of StAR expression without corresponding elevation in progesterone production. This is probably due to the fact that in the immortalized granulosa cell lines no basal levels of the cytochrome p450scc, the activity of which is obligatory for the conversion of cholesterol to pregnenolone, can be detected [32]. This notion is supported by our preliminary findings that in primary rat granulosa cells obtained from pre-ovulatory follicles and do contain p450scc, PD98059 by itself increased progesterone production. On the other hand, MEK inhibitors do synergies with gonadotropin/cAMP stimulation of steroidogenesis because of the de-novo synthesis of the cytochrome p450scc, which is stimulated by gonadotropin/cAMP in the granulosa cell lines [17,32].

### 3.5. Subcellular localization of the overexpressed StAR

**Figure 6:**
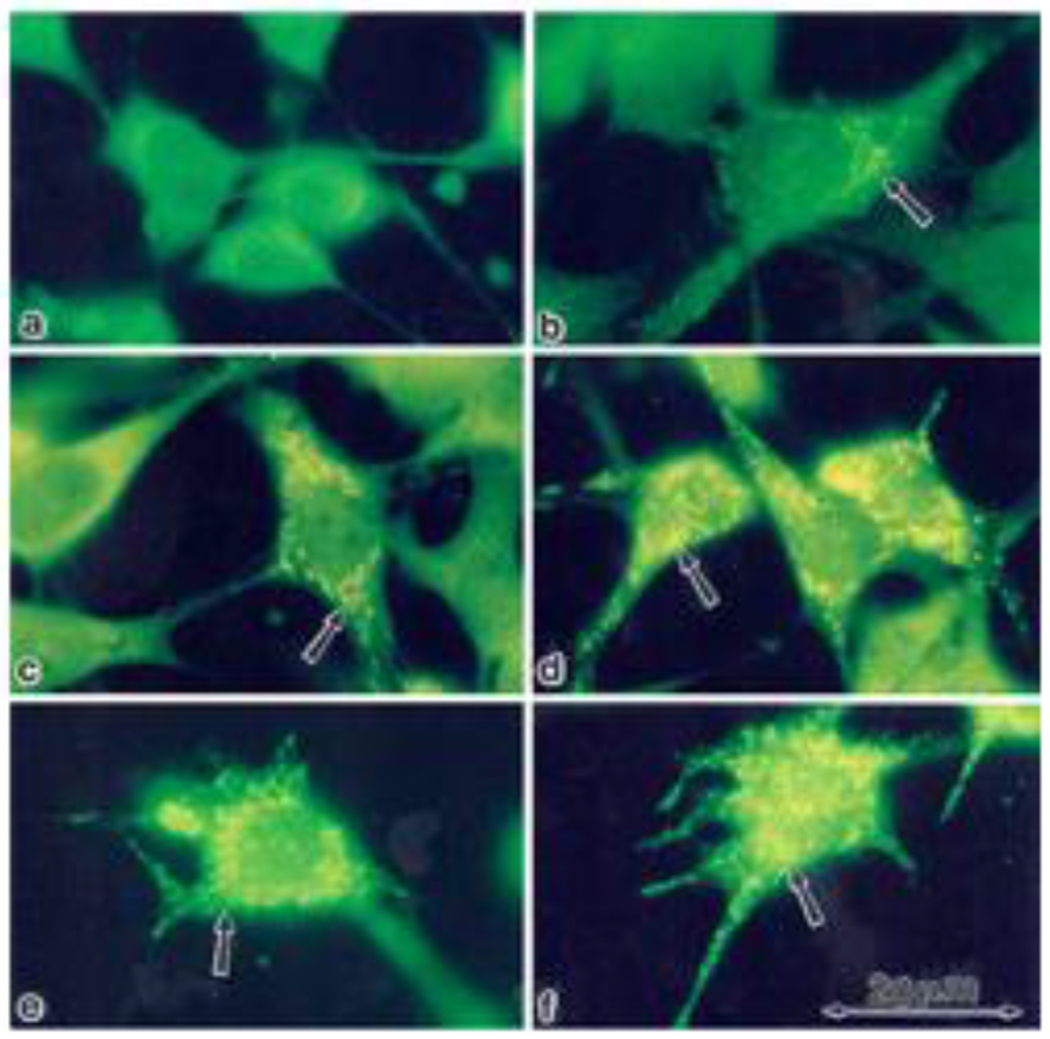
Subcellular localization of StAR upon induction with FSH and PD98059. Immunofloresence of cells stained with anti-StAR antibodies followed by goat anti-rabbit IgG conjugated to fluorescein, Subconfluent rFSHR-17 cells were stained with anti-StAR antibodies prior to or following PD98059, FSH and forskolin stimulation. a - no treatment; b - 24 h incubation with PD98059 (25 μM); c - 24 h incubation with LH (3 iu/ml); d - 24 h incubation with PD98059 (25 μM) and LH (3 iu/ml); e - 24 h incubation with 8-br-cAMP (50 μM; f - 24 h incubation with PD98059 (25 μM) and 8-br-cAMP (50 μM). Florescence microscopy x 1330. The arrow indicates StAR staining in the mitochondria.

In order to examine whether the enhancement of StAR expression by PD98059, gonadotropins and cAMP-elevating agents is mainly located in mitochondria [27], we stained rFSHR-17 cells with anti-StAR antibodies prior or following PD98059, FSH and forskolin stimulation (Figure 6). In non-stimulated cells, StAR could not be detected in mitochondria (a). In contrast, clear elevation in mitochondrial StAR was evident following 24 h of treatment with PD98059 (b). LH clearly increased the StAR content in the mitochondria (c) while PD98059 dramatically increased mitochondrial StAR content (d). 8 Br cAMP augmented StAR levels (e) while PD98059 further enhanced StAR content in the mitochondria (f). Thus, the immunocytochemical observations confirmed the data obtained by Western blot on the elevation of StAR expression by PD98059.

### 3.6. Gonadotropin-induced ERK activation and StAR production are mediated by PKA

**Figure 7:**
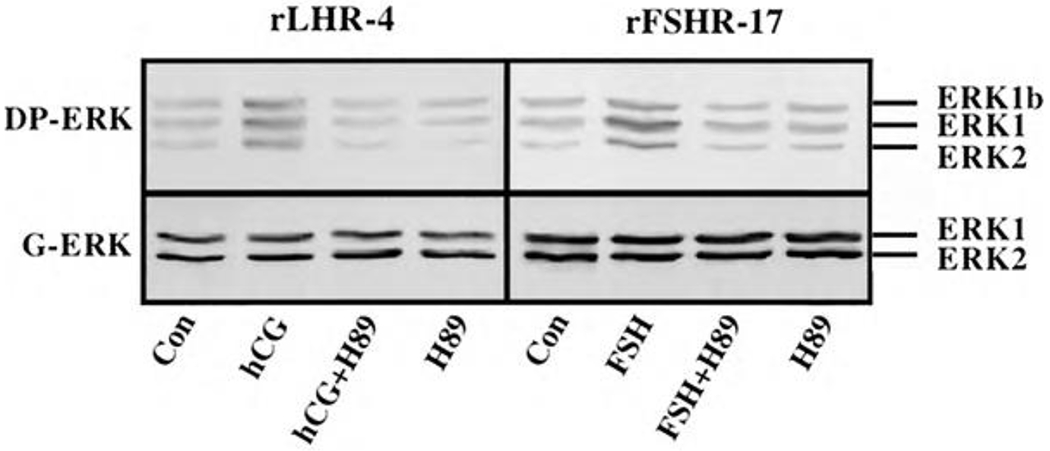
Effect of H89 on activation of ERK in gonadotropin-treated rLHR-4 and rFSHR- 17 cells. rLHR-4 or rFSHr-17 cells were serum-starved for 16 h and then stimulated with the appropriate gonadotropins (3 iu/ml, 10 min) with or without the PKA inhibitor, H89 (15 min pre-stimulation, 3 μM). Cytosolic extracts (50 μg) were subjected to immunoblotting with DP- ERK (upper panel) or with anti-general ERK antibody (G-ERK, second panel). The ERK2, ERK1, and ERK1b are indicated.

**Figure 8:**
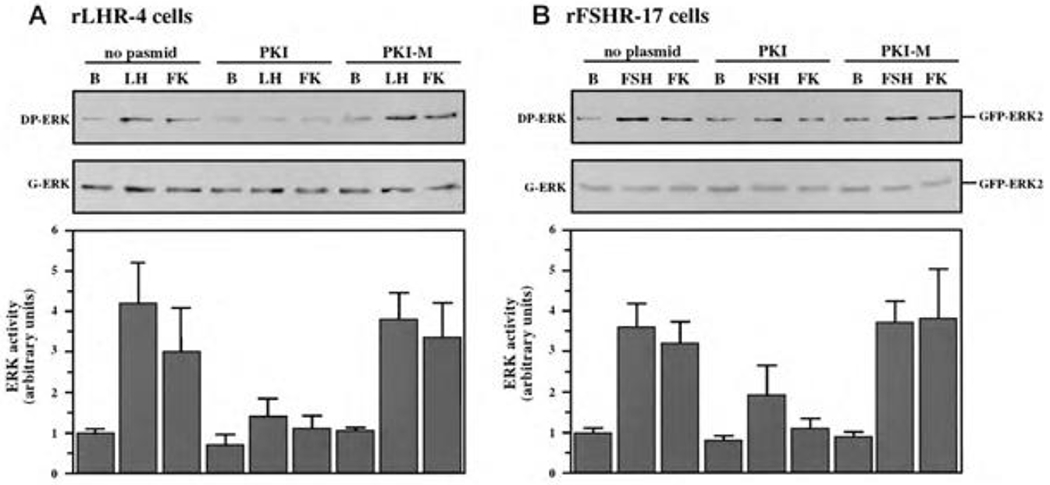
Effect of PKI on ERK-activation by gonadotropins and by forskolin. rLHR-4 (A) and rFSHr-17 (B) cells were transfected with pGFP-ERK2 alone (no plasmid) or co-transfected with pGFP-ERK2 together with RSV-PKI (PKI), and RSV-PKI mutant ((PKI-M, which is inactive PKI). After transfection the cells were treated as described under Material and Methods for 18 h and then stimulated with FSH (3 iu), forskolin (FK, 50 μM) for 10 min or left untreated (B). The cells were then harvested, and cytosolic extracts were subjected to western blot analysis with anti-DP-ERK and anti-C16 antibodies (G-ERK). The 70 kDa band which represent GFP-ERK2 is shown in the upper panels. Densitometric scanning of the DP-ERK lanes (arbitrary units) were used as a measure for ERK activity (bar-graphs, bottom panels). The results in the bar graphs are average and standard errors of three experiments.

**Figure 9:**
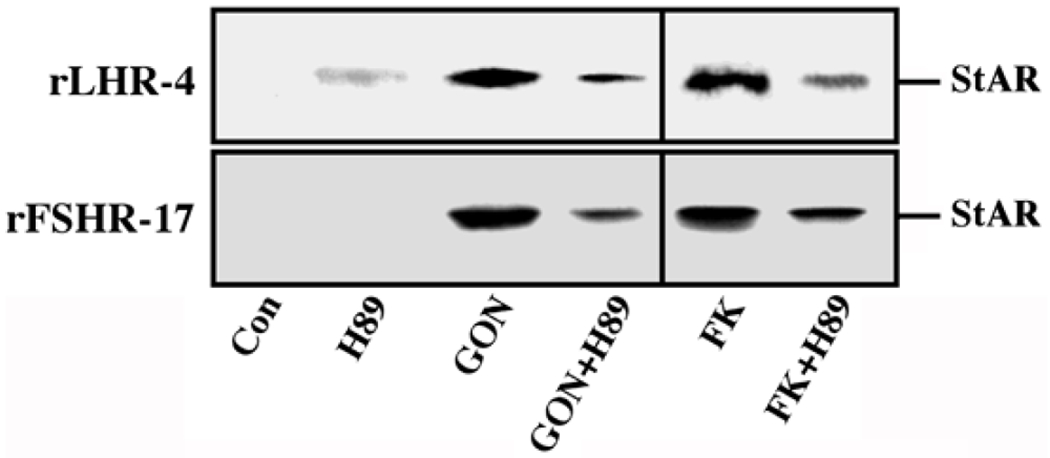
Effect of H89 on the expression of StAR in gonadotropin-treated rLHR-4 and rFSHR-17 cells. rLHR-4 or rFSHr-17 cells were serum-starved for 16 h and then stimulated with the appropriate gonadotropins (3 iu/ml, 10 min) with or without the PKA inhibitor, H89 (15 min pre-stimulation, 3 μM). Cytosolic extracts (50 μg) were subjected to immunoblotting with anti-StAR antibody. The arrow indicates mature StAR protein at 32 kDa.

**Figure 10:**
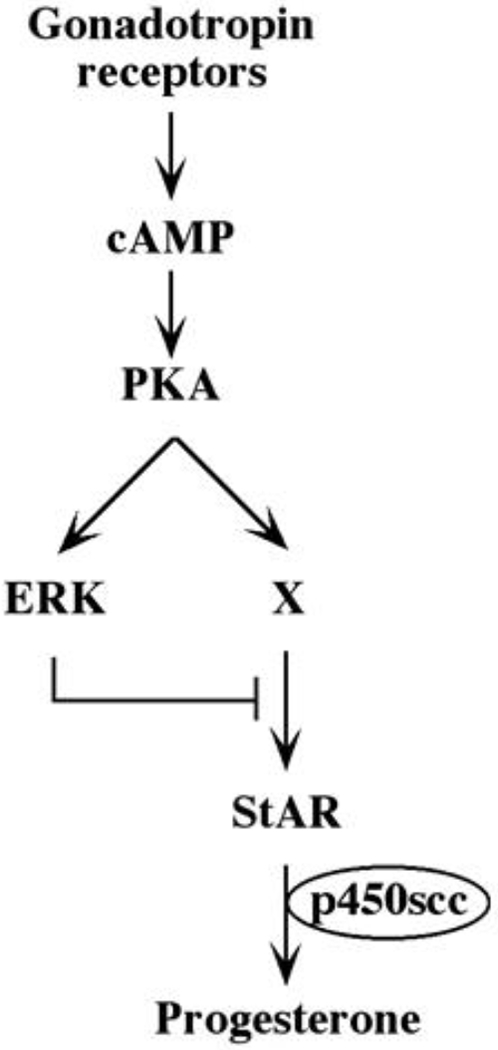
Schematic representation of signaling pathways controlling gonadotropin-induced steroidogenesis.

Although we showed that an elevation of cAMP is sufficient to activate ERK, it was not clear whether cAMP and PKA are the major mediators of the gonadotropin-generated signaling to ERK. Therefore, we used H89, which is a potent and selective inhibitor of PKA to study the involvement of cAMP/PKA in the activation of ERK in rLHR-4 and rFSHR-17 cells. The addition of 3 μM of H89 15 min prior to gonadotropin stimulation did not change the basal activity of the three ERKs, but completely abrogated the induction of ERK by hCG in rLHR-4 cells and by FSH in rFSHR-17 cells (Figure 7). As expected, ERK activation by forskolin and 8-Br-cAMP in both cell lines was also inhibited by H89 (data not shown), indicating that ERK activation is mediated mainly by PKA and probably not via the cAMP-GRF [33]. In order to further verify the involvement of PKA in the activation of ERK by gonadotropins we co-expressed GFP-ERK2 [21] together with the potent PKA inhibitor PKI or its inactive mutant (PKI mutant [20]). Specific activation of ERK in the transfected cells was measured by the incorporation of phosphate into the activation loop of the GFP-ERK2 with anti-DP ERK antibodies. As observed with H89, inhibition of PKA with PKI significantly inhibited ERK activation by gonadotropins and by forskolin (Figure 8). Taken together, these results clearly indicate that the activation of ERK by gonadotropin in the cell lines examined is mostly PKA dependent. We then examined whether StAR activation is mediated by PKA alone. Indeed, when the PKA inhibitor H89 was added to rLHR-4 and rFSHR-17 cells it significantly inhibited hCG- and FSH-stimulated StAR expression (Figure 9). As expected, H89 also attenuated forskolin- induced StAR expression (Figure 9), indicating that StAR production is regulated by PKA in the cell lines examined. As expected, progesterone production was also significantly inhibited by the H89 inhibitor (data not shown), indicating that the processes examined may function mainly downstream of PKA. However, progesterone production most probably lies downstream of PKA and of StAR, whereas ERK, although activated by PKA, serves as a negative regulator of this pathway due to its suppression of StAR (Figure 10).

## 4. Discussion

In this manuscript we demonstrate a mechanism for cross-talk between two signaling pathways, the cAMP/PKA and the ERK cascade in a Gs-induced system. The interaction between these two cascades has been extensively studied in several cellular systems over the past few years [34]. In many systems, such as in EGF-stimulated Rat1 fibroblasts [35] or PDGF-stimulated human arterial smooth muscle cells [36], it was shown that cAMP inhibit the activation of the ERK cascade. This inhibition seems to occur by either inhibitory phosphorylation of Raf-1 [35] or by activation of the small GTPase, Rap-1, which compete with Ras for the activation of Raf-1 [37]. In other cell systems such as NGF-stimulated PC12 cells, cAMP not only does not inhibit the ERK cascade, but in fact activates it to induce various mitogenic or differentiation processes. One mechanism that activates the ERK cascade by PKA includes the activation of the cAMP responsive guanine-nucleotide exchange factors for the small GTPase Rap1, Epac1 and Epac2. Upon binding of cAMP, these components rapidly activate Rap1, which then promotes the activation of B-Raf (but not Raf-1) and the rest of the ERK cascade [33]. However, in the rLHR-4 and rFSHR-17 cells used in our study, the activation of ERK seems to be downstream of PKA, indicating that the Epac’s are probably not involved in the ERK activation. This pathway may then involve an activation of the Rap-1 GTPase by PKA which causes the tight association with B-Raf and induction of the ERK cascade. Recently, it was shown that activation of ERK by cAMP in the brain might occur via a cAMP-responsive STE-20-like kinase, MST3b [38]. Although this specific isoform does not seem to be expressed in granulosa cells, it is possible that another MAP4K or MAP3K is involved in the transmission of PKA signals to ERK in the rLHR-4 and rFSHR-17 cells.

The involvement of PKA in gonadotropin-dependent ERK activation is demonstrated in the present work both by pharmacological means using PKA inhibitor H89, and by genetic means, i.e. transfection of cells with plasmid encoding for PKI. The data using both methods are in good agreement that PKA plays a major role in transducing gonadotropin signaling towards ERK. Nevertheless, it should be noted that although PKI completely suppressed forskolin-induced ERK activation, it did not completely inhibit the gonadotropin-induced ERK activation. Therefore, it is quite possible that the gonadotropin receptors are using other G proteins or the Gβγ subunits of Gs protein to activate the ERK cascade, as was observed for other receptors and cell types (reviewed in [23,39]). Interestingly we recently found that basic FGF suppresses progesterone production in the granulosa cell lines (data not shown), which would suggest that there may be alternative pathways in these cells that suppress steroidogenesis via gonadotropin/cAMP independent ERK cascade. Cooperation between the cAMP/PKA and the ERK pathways has been demonstrated in several cells. For example, it was shown that cAMP causes sustained activation of the ERK cascade, which is important for neurite outgrowth in PC-12 cells [40]. In human cyst epithelial cells, elevation of cAMP causes a mitogenic response that is mediated primarily by the ERK cascade [41]. However, it was also shown that cAMP-induced processes might contribute to a late down-regulation of ERK-mediated processes. An example of this interaction of the PKA-induced CPG16 kinase, which seems to partially inhibit the activity of the transcription factor CREB [42], suggests its involvement in the down-regulation of cAMP- and ERK cascade- induced transcription. In contrast to this type of interaction, we show here that the activation of processes downstream of PKA may also be inhibited by an ERK-mediated mechanism. The inhibition of cAMP-induced progesterone production could occur at the level of phosphorylation-dephosphorylation of proteins that play a role in the steroidogenic pathway. In the present study we examined the expression of StAR protein, which is known to be phosphorylated on serine or threonine residues [43]. Although StAR phosphorylation may play a role under distinct circumstances, it did not seem to correlate with the induction of PKA or ERK cascades and we could not detect any direct phosphorylation of StAR by ERK (data not shown). However, we did observe an inverse correlation between ERK activity and StAR expression in the mitochondria. The blockade of ERK activity caused an elevation in the amount of StAR protein, while activation of ERK by TPA reduced StAR expression in granulosa cells. Therefore, it is probable that the two cascades interact to regulate StAR gene transcription, the primary mechanism for regulating StAR expression in granulosa cells [44].

Several transcription factors including steroidogenic factor-1 (SF-1), C/EBP and the negative regulator DAX-1 [45,46,47] participate in the transcriptional regulation of this gene. StAR gene transcription is probably driven by the SF-1 and C/EBP downstream of PKA, but it is unlikely that these components participate in the downregulation of StAR expression via the ERK cascade, because both have been shown to be stimulated by ERK [48,49]. Therefore, it is possible that the negative regulation of StAR expression occurs at the level DAX-1 or some yet to be identified transcription factor. Alternatively, StAR expression could be controlled by induction of potent phosphatases that abolish both the PKA and ERK phosphorylation of SF-1 and C/EBP, or induce a proteolytic system that reduces the half-life of the StAR protein. Another explanation for the mechanism by which ERK can inhibit steroidogenesis could be its involvement in desensitization of the gonadotropin receptors. Prolonged incubation of granulosa cells with gonadotropic hormones has previously been shown to cause desensitization of the cells to further stimulation, which is characterized by downregulation of cAMP formation as well as of steroidogenesis [9]. Moreover, it has previously been demonstrated that the ERK cascade could activate G-protein coupled receptor kinase 2 [50], which in turn induces down-regulation of seven transmembrane receptors. However, it is unlikely that this is the mechanism in our case, because the inhibitory effects of ERK were demonstrated when cells were stimulated by cAMP. Since this activator can bypass the receptor to directly activate PKA, most of the inhibitory signals are probably receptor-independent. Nevertheless, under physiological conditions, the gonadotropins play a key role in modulation of ERK activity. Moreover, activation of ERK can explain the mitogenic signals exerted by FSH during folliculogenesis. It is known that unlike the initiation of steroidogenesis, which is proportional to the duration and extent of cAMP production, full activation of ERK can be achieved as a consequence of even modest increases in intracellular cAMP. This amplification occurs due to a switch-like mechanism of the ERK cascade, which allows a strong signaling output even by weak extracellular signals [51]. The stronger activity of ERK, which functions downstream of cAMP, may explain the suppression of steroidogenesis upon weak gonadotropic signals, which lead to steroidogenesis. The effects can be modulated by distinct subcellular localizations as well [52]. These two situations may explain the low levels of steroidogenesis induced by dghCG, able to induce only weak signals by the GnRHR. In conclusion, the present study shows that activation of cAMP/PKA signaling by gonadotropins not only induces steroidogenesis, but also activates down-regulation machinery that involves the ERK cascade. This potent down-regulation machinery inhibits the gonadotropin-induced steroidogenic pathway by mechanisms that are different from the well-characterized receptor desensitization mechanisms. Activation of the ERK cascade downstream of PKA, in turn regulates the level of StAR expression, which is probably the key participant in these down-regulation processes. Thus, PKA not only mediates gonadotropic-induced steroidogenesis, it also activates the downregulation mechanism that can silence steroidogenesis under certain conditions. Moreover, our findings raise the possibility that activation or inhibition of ERK by other pathways could be an important mechanism for diminution or amplification of gonadotropin-stimulated steroidogenesis. This could contribute to functional luteolysis, a process in which leutinized granulosa cells show reduced sensitivity to LH despite maintenance of LH receptor or to up-regulation of the steroidogenic machinery during luteinization of granulosa cells (reviewed in [53]).

## Abbreviations

CG: Chorionic gonadotropin
DMEM: Dulbecco’s modified Eagle’s medium
FSH: Follicle-stimulating hormone
dghCG: Deglycosylated hCG
ERK: Extracellular signal-regulated kinase
hCG: Human chorionic gonadotropin
MAPK: Mitogen-activated protein kinase
MBP: Myelin basic protein
LH: Luteinizing hormone
PKA: Protein kinase A
PKI: Protein kinase inhibitor
StAR: Steroidogenic Acute Regulatory Protein
TPA: 12-O-tetradecanoylphorbol-13-acetate
8-Br-cAMP: 8-bromo-cyclic AMP
